# When Numbers Lie: A Case Report of Falsely Low Hemoglobin Due to Preanalytical Error Leading to Unnecessary Interventions

**DOI:** 10.7759/cureus.97937

**Published:** 2025-11-27

**Authors:** Adam Zoubi

**Affiliations:** 1 Internal Medicine, CHRISTUS Spohn Hospital, Corpus Christi, USA

**Keywords:** blood sample dilution, false-positive anemia, laboratory diagnostic error, laboratory medicine quality improvement, preanalytical laboratory error, sample collection error, spurious laboratory results

## Abstract

False laboratory values can trigger extensive and unnecessary diagnostic and therapeutic workups. We report a case of falsely diagnosed severe anemia that led to blood transfusions and a comprehensive evaluation, which included aggressive intervention as an endoscopy, before being traced back to a preanalytical sample collection error. Proper phlebotomy technique and education, clinical correlation, and sometimes confirmatory labs can help avoid scenarios like the one presented in this case report.

## Introduction

Making medical decisions, especially during the first encounter at the emergency department and admission encounter, is heavily influenced by laboratory workup, yet lab workup is not free of error. Errors may occur in the preanalytical phase (test selection, patient preparation, specimen collection, labeling, transport, and processing), the analytical phase (measurement), or the post‑analytical phase (reporting and interpretation). Preanalytical errors are particularly important because they affect the integrity of the specimen before it ever reaches the analyzer. Preanalytical errors are commonly defined as any mistakes that occur between the clinician’s test request and the start of the analytical process. Typical examples include incorrect patient identification, use of the wrong collection tube, inadequate sample volume, clotted or hemolyzed samples, sample dilution from intravenous fluids, improper storage, and delays or extremes in transport temperature. Although many preanalytical errors are intercepted by laboratory quality systems and result in specimen rejection rather than result release, those that reach clinicians can directly contribute to misdiagnosis, inappropriate transfusion or procedures, prolonged length of stay, and increased costs. Emergency and acute care settings are especially vulnerable to preanalytical error. High patient volume, time pressure, frequent use of temporary or trainee staff, and the need for rapid vascular access all increase the risk of suboptimal specimen collection, such as drawing blood from running IV lines, failing to discard an adequate waste volume, or submitting insufficient or clotted complete blood count (CBC) samples. Studies analyzing preanalytical errors show that most of these errors occur in the emergency department [1]. An estimated 60-70% of medical decisions rely on lab results. However, the laboratory testing process (including preanalytical, analytical, and post‑analytical phases) is vulnerable to error at multiple steps. Overall laboratory error rates have been estimated at 0.012-0.6% of all test results, but the majority of these errors arise before the sample is ever analyzed [2]. Although many preanalytical errors are intercepted by laboratory quality systems, those that reach the clinician can lead to misdiagnosis, unnecessary procedures, inappropriate transfusions, prolonged length of stay, and increased healthcare costs [3]. These laboratory errors contribute to diagnostic errors, which account for 6-17% of hospital adverse events [4], and 10% of all patient deaths were attributed back to diagnostic error in a multi-decade postpartum examination research. In the case discussed below, our patient was involved in this error and was initially admitted for an acute normocytic anemia workup and treatment. It illustrates how discordant laboratory values can distort the clinical picture, initiating a cascade of unnecessary interventions, and underscores the need for systematic safeguards such as repeat testing of critical outliers, standardized phlebotomy training, and robust communication between clinical and laboratory teams.

## Case presentation

A 33-year-old female with a past medical history of a heart murmur since childhood and a cholecystectomy, not on any medications or over-the-counter supplements, presented with exertional shortness of breath that had started to occur at rest, but denied chest pain. The patient also reported a two-day history of worsening of her chronic heartburn. She denied any bloody vomitus, melena, bloody stool, or gross hematuria. The patient reported a regular menstrual cycle and denied heavy menstruation. She also denied bruising, bleeding from the gums when brushing her teeth, or nosebleeds.

The patient denied any family history of major health issues, cancer, autoimmune disease, or bleeding disorders. She denied tobacco, alcohol, or recreational drug use.

Physical examination showed vital signs included blood pressure of 107/72 mmHg, heart rate of 84 beats/min, respiratory rate of 17 breaths/min, temperature of 98.8°F, and oxygen saturation of 96% on room air. She was alert and oriented and speaking in full sentences and did not appear in acute distress. There was no orthostatic hypotension or tachycardia on standing. Peripheral perfusion was preserved with warm extremities and capillary refill <2 seconds. Mucous membranes and conjunctiva were not pale on examination, and there was no evidence of jaundice or petechiae. Abdominal exam was notable for mild epigastric tenderness; digital rectal exam was negative for bloody or melenic stool. A mild systolic heart murmur was auscultated, but otherwise the exam was unremarkable and negative for any signs of bleeding or bruising.

A month prior to admission, the patient had seen her primary care physician (PCP) to establish outpatient care. Table 1 presents her laboratory findings at that time

**Table 1:** Prior primary care physician (PCP) visit lab results (1 month before admission)

Test	Result	Reference Range/Normal Values
Hemoglobin (Hgb)	11.2 g/dL	12–16 g/dL (female)
White Blood Cells (WBC)	8.2 × 10³/μL	4.5–10.0 × 10³/μL
Platelets (Plt)	189 × 10³/μL	150–400 × 10³/μL
Ferritin	71 ng/mL	13–150 ng/mL

Table 2 presents her lab work at the Emergency Department (ED) day of admission.

**Table 2:** Laboratory results at the Emergency Department (at presentation)

Test	Result	Reference Range/Normal Values
Hemoglobin (Hgb)	5 g/dL	12–16 g/dL (female)
Mean Corpuscular Volume (MCV)	88 fL	80–100 fL
Red Blood Cells (RBC)	1.6 × 10⁶/μL	3.8–5.1 × 10⁶/μL
White Blood Cells (WBC)	3.6 × 10³/μL	4.5–10.0 × 10³/μL
MCHC	32.1 g/dL	32-37 g/dL
RDW	12.7 %	10.5-15.5%
Platelets (Plt)	133 × 10³/μL	150–400 × 10³/μL
Peripheral Smear	Normal RBC morphology; no blasts or schistocytes	—
Haptoglobin	<8 mg/dL	30–200 mg/dL
Total Bilirubin	0.8	0.2–1.2 mg/dL
Lactate Dehydrogenase (LDH)	162	140–280 U/L
Iron Studies	Not done (due to transfusion)	-
Vitamin B12	412	200–900 pg/mL
Folate	11	2.7–17 ng/mL
HIV	Negative	Negative
ANA	Negative	Negative
β-HCG	Negative	Negative
Hepatitis Panel	Negative	Negative
Direct Coombs Test	Negative	Negative
Stool Occult Blood	Negative	Negative

Lactic acid was within normal limits at 1.2. Our Lab department did not report to the physician/nursing staff regarding any analyzer error or red flags regarding the sample, but reported Hgb of 5 as a critical value. Despite the discrepancy between the patient’s hemodynamic stability and the severely low hemoglobin value, the ED/admission team prioritized prompt transfusion and upper endoscopy to avoid missing a potentially life-threatening gastrointestinal (GI) source of bleeding, accepting the lab result as valid at the time, and two units of packed red blood cells were transfused.

Patient intake status changed to nothing by mouth (NPO), and empiric IV Protonix was started. Gastroenterology was consulted, and the patient underwent EGD, which did not show a source of bleeding. Table 3 presents her repeat laboratory work the next morning.

**Table 3:** Laboratory results post transfusion/inpatient

Test	Result	Reference Range/Normal Values
Hemoglobin (Hgb)	14.1 g/dL	12–16 g/dL (female)
Mean Corpuscular Volume (MCV)	89 fL	80–100 fL
Red Blood Cells (RBC)	4.64 × 10⁶/μL	3.8–5.1 × 10⁶/μL
White Blood Cells (WBC)	6.7 × 10³/μL	4.5–10.0 × 10³/μL
Platelets (Plt)	253 × 10³/μL	150–400 × 10³/μL

Due to the negative workup, a preanalytical laboratory error was suspected. The initial sample showing Hgb 5 g/dL was traced to a sample collected from a peripheral IV access instead of routine venipuncture, as reported by the nurse who did the blood collection. The patient was discharged in stable condition with a follow-up appointment with her PCP and established cardiologist.

## Discussion

Clinical reasoning often begins with laboratory data, which heavily influence the diagnoses made, especially in the emergency department or during admission encounters where quick decision-making is essential. However, laboratory results are not always error-free; they can mislead even experienced clinicians who are racing against time to deliver care without delay. Being aware of the rate of laboratory errors can remind clinicians that labs that do not correlate with the clinical picture should trigger suspicion before making medical decisions based completely on these labs.

Our case underscores this vulnerability, which was treated as presumed severe anemia, which prompted extensive diagnostic workup, and was later traced to a specimen collection error.

This case is noteworthy because it demonstrates the real-world impact of preanalytical errors on clinical decision-making and patient safety. While most clinicians are aware that lab error is possible, it is rarely the first consideration when facing a critically abnormal result. In practice, such errors can trigger a cascade effect, a chain reaction where an initial test triggers a series of subsequent events, with each one causing the next and often amplifying the overall impact of testing. This can lead to unnecessary procedures, blood product utilization, prolonged hospitalization, and increased healthcare costs, ultimately resulting in low-value care. Such care provides little or no benefit, can potentially cause harm, and wastes healthcare resources, all of which are preventable if clinical context and laboratory plausibility are carefully reconciled. Beyond diagnostic confusion, preanalytical errors consume substantial resources. One analysis estimated that up to 75% of all laboratory errors occur before analysis and that roughly one‑quarter of these have the potential for direct clinical harm or delayed diagnosis [3]. Our case exemplifies this, as a single erroneous result led to hospital admission, blood product utilization, an urgent endoscopy, and significant patient anxiety.

Laboratory errors have a reported frequency of 0.012-0.6% of all test results [5]. In a two-year study done in Saudi Arabia [1] evaluating 55,345 laboratory tests, an overall rate of 12.1% (6,705) was determined to be preanalytical errors. The occurrence of these errors was highest in the emergency department (21%). These errors occur before the sample is analyzed - during collection, handling, labeling, or transport. Common causes include hemolysis (most common), sample dilution from IV fluids, improper tube filling or mixing, and incorrect patient identification.

Similar rates have been reported globally [6], suggesting a consistent pattern in high-throughput, fast-paced clinical settings where phlebotomy protocols may be inconsistently applied. Obtaining blood from an intravenous line used for IV fluids can dilute the sample; 2.2% of all samples were rejected due to sample contamination with IV fluids [7], giving artificial results such as low red cell, platelet, and white blood counts. For hemoglobin measurement, dilutional error is a known cause of false-positive anemia results [8]. As in our case, the initial specimen was drawn from a peripheral IV line, revealing a discrepancy between the patient’s clinical stability and the alarming laboratory value.

Another cross-sectional study [9] noted that the complete blood count (CBC) rejection rate was 5% during that study; 16.35% of the rejected samples were due to diluted samples. Interestingly, the trend and origin of these diluted samples were linked to a specific hospital/period of time, when that hospital hired new trainee nursing personnel. Again, emphasizing the importance and necessity of proper phlebotomy education. A large percentage of errors have been reported frequently [6] in an overview study with 46-68% rejection due to sample collection/quality and methods. Recognizing these errors is important in order to solve the root cause of these issues, as done in a large study in China [6], resulting in decreased numbers of errors recognized in a month period analysis of 10.1 million samples collected, where preanalytical errors were significantly reduced to 0.11%.

This case emphasizes a critical aspect of modern medicine: the responsibility to interpret the increasing amount of available data for a patient within the context of the clinical picture. Laboratory testing has evolved, but human oversight for possible errors along the way remains unavoidable in some cases. The absence of symptomatic anemia, normal vital signs, and lack of GI bleeding evidence should prompt reconsideration of previous lab values before initiating transfusions or aggressive interventions like endoscopy.

This case highlights a multidisciplinary issue, including phlebotomy training, clear communication between nursing, laboratory, and physician teams, and prompt retesting of discordant results, which are key safeguards. Many institutions now encourage reflex repeat testing for critical outliers prior to result release, and our case supports implementing such measures to reduce preventable harm.

From a diagnostic safety perspective, this case illustrates a classic diagnostic cascade: an unexpectedly critical laboratory value triggered a series of downstream interventions: type and crossmatch, red blood cell transfusion, and urgent esophagogastroduodenoscopy that did not ultimately reveal pathology or alter the patient’s long‑term care. Similar cascades have been described after incidental imaging or laboratory findings that are of limited clinical significance yet prompt extensive follow‑up, exposing patients to procedure-related risks, anxiety, and opportunity costs. In the terminology of low‑value care, these interventions offered little net benefit once considered in light of the patient’s reassuring hemodynamics and negative initial evaluation, and they consumed limited healthcare resources that might otherwise have been directed toward patients with clear indications for transfusion or endoscopy.

Importantly, the clinicians’ actions were reasonable in real time, given the information available and the high stakes of missing catastrophic GI bleeding. This highlights that the solution is not simply to “do less,” but to build system‑level safeguards - such as automatic reflex repeat testing of discordant critical values and rapid clinician‑laboratory communication - that allow teams to distinguish false from true emergencies before committing patients to invasive procedures.

Our findings align with prior literature [1,10] that advocates for routine education on preanalytical variables for clinical and laboratory staff, repeating tests of critical results that are inconsistent with the clinical picture when findings do not correlate with the patient’s presentation, and enhancing feedback loops between the laboratory and care team to identify and correct recurring collection errors.

## Conclusions

This case highlights the importance of clinical correlation when interpreting laboratory results. A lab value that appears inconsistent with the patient’s presentation should raise the consideration for verification rather than immediate aggressive interventions. We propose four practical safeguards: (1) reflex repeat testing of discordant critical values - laboratories and EDs should adopt protocols in which unexpectedly critical results that conflict with the clinical picture prompt immediate repeat sampling before blood products are released or invasive procedures are initiated; (2) standardize phlebotomy education for nursing staff and phlebotomy staff to emphasize correct tube selection, adequate sample volume, and avoidance of blood draws from IV lines or recently infused sites unless explicitly ordered; (3) mandate nursing/phlebotomy staff to label blood withdraw location and the blood obtaining method used; and (4) include automatic instructions with each lab ordered through electronic nedical record (EMR) directions for appropriate phlebotomy practices, for example, not to draw blood from IV access through which IV fluids is running. These steps, if taken or implemented, can help avoid a similar cascade of events that lead to further unnecessary workup, which did not change the management plan for our patient. We recommend the following approach to discordant hemoglobin results in a hemodynamically stable patient as a practical algorithm: (1) pause and reassess the patient - confirm vital signs, symptoms, physical exam (including signs of bleeding), and basic markers of perfusion (e.g., mental status, lactate); (2) hold non‑urgent interventions - if there is marked mismatch between the lab value and the bedside assessment, defer transfusion and invasive procedures unless the patient is unstable; (3) immediately repeat sampling from a clean site - obtain a new CBC from a venepuncture in the contralateral limb, avoid drawing from IV lines whenever possible; if unavoidable, strictly follow protocols (stop infusion, discard adequate waste volume, document site and method); (4) perform laboratory cross‑checks - ask the laboratory to review HIL (hemolysis, icterus, and lipemia) indices, mean corpuscular hemoglobin concentration (MCHC), red cell distribution width (RDW), and CBC histograms for flags suggesting dilution, clots, agglutination, or other interferences; (5) consider a point‑of‑care or alternate Hb method (e.g., HemoCue or a different analytical principle) if discordance persists; (6) compare with prior results (delta check) - large, abrupt changes from recent baseline (e.g., >3 g/dL Hb drop) should trigger suspicion for error and a delta‑check workflow; and (7) act on confirmed results - if the repeat Hb confirms true severe anemia and the clinical picture is compatible, proceed with guideline‑based transfusion and further workup. If the repeat Hb is near baseline and alternative interferences have been reasonably excluded, treat the index value as spurious, notify the laboratory, and consider a local quality improvement review.

In addition to technical safeguards, this case underscores the need for structured, not merely informal, communication between the laboratory and the clinical team when a critical value does not fit the bedside examination. For physiologically implausible results, we recommend formal policies that require direct telephone notification from the laboratory technologist to the treating clinician; immediate venipuncture redraw from a clean site; and a brief joint review of the result, indices, and clinical findings before blood products are released or invasive procedures are undertaken. In our patient, such a lab-ED conversation at the time the hemoglobin of 5 g/dL was reported would likely have triggered a redraw and prevented both transfusion and urgent endoscopy. Embedding these steps into institutional critical‑value and discordant‑result pathways aligns with broader patient‑safety frameworks that emphasize shared responsibility across teams and help ensure that future patients are protected from similar cascades triggered by spurious laboratory values.
